# Synergistic enhancement of clustered regularly interspaced short palindromic repeats (CRISPR)/CRISPR-associated protein 9 -mediated gene editing in porcine zygotes through combined lipofection and electroporation of cationic lipid-packaged ribonucleoproteins

**DOI:** 10.14202/vetworld.2025.3806-3814

**Published:** 2025-12-10

**Authors:** Qingyi Lin, Takeshige Otoi, Oky Setyo Widodo, Theerawat Tharasanit, Kaywalee Chatdarong, Zhao Namula, Maki Hirata, Aya Nakai, Yuichiro Nakayama, Megumi Nagahara, Fuminori Tanihara

**Affiliations:** 1 Bio-Innovation Research Center, Tokushima University, 779-3233, Tokushima, Japan; 2 Division of Animal Husbandry, Faculty of Veterinary Medicine, Universitas Airlangga, 60115, Surabaya, Indonesia; 3 Department of Obstetrics, Gynaecology and Reproduction, Faculty of Veterinary Science, Chulalongkorn University, 10330, Bangkok, Thailand; 4 Department of Veterinary Medicine, College of Coastal Agricultural Sciences, Guangdong Ocean University, 524091, Zhanjiang, China

**Keywords:** cationic lipid, CRISPR/Cas9, electroporation, genome-editing efficiency, lipofectamine, porcine zygote, xenotransplantation

## Abstract

**Background and Aim:**

Genetically engineered pigs are invaluable biomedical models for xenotransplantation and the study of human diseases. Although electroporation (EP) and lipofection are individually effective for clustered regularly interspaced short palindromic repeats (CRISPR)/CRISPR-associated protein 9 (Cas9) ribonucleoprotein (RNP) delivery, their combined application in porcine embryos has not been systematically evaluated. This study aimed to determine whether packaging Cas9-guided RNA complexes in cationic lipids enhances EP-mediated gene editing efficiency without compromising embryonic development.

**Materials and Methods:**

Porcine zygotes with their zona pellucida removed were edited using RNPs targeting beta-1,4-N-acetyl-galactosaminyl transferase 2 (*B4GALNT2*) or growth hormone receptor (*GHR*) genes. Four treatment groups were tested: (1) EP with RNPs (EP), (2) EP with lipofectamine-packaged RNPs (EPL), (3) transfection with lipofectamine-packaged RNPs before EP (TL + EPL), and (4) EP followed by lipofection (EPL + TL). Blastocyst formation was evaluated morphologically, and mutation rates were assessed by Sanger sequencing followed by tracking of indels by decomposition (TIDE) analysis.

**Results:**

Blastocyst formation rates were comparable across all treatments, indicating that lipofectamine packaging and EP caused no detectable cytotoxicity. For *B4GALNT2*, no mutations were induced by EP alone, whereas TL + EPL treatment significantly increased total and mosaic mutation rates (p < 0.05). For *GHR*, the total mutation and mosaic mutation rates were likewise higher in TL + EPL compared with EP, although mutation efficiency (indel percentage per edited embryo) remained unchanged. These results suggest that pre-EP lipofection promotes RNP uptake by facilitating lipid-membrane interactions that are potentiated by subsequent membrane destabilization through EP.

**Conclusion:**

Packaging RNPs in cationic lipids and applying sequential lipofection followed by EP significantly enhances CRISPR/Cas9-mediated mutagenesis in porcine zygotes without affecting developmental competence. This dual-delivery approach provides a simple, reproducible, and low-toxicity workflow for generating gene-edited embryos, with potential applicability to large-animal biomedical models.

## INTRODUCTION

The CRISPR/Cas9 system, consisting of the Cas9 nuclease and a single guide RNA, has become a versatile and powerful platform for precise genome modification across multiple species [1]. Among livestock, pigs are particularly important in biomedical research due to their close anatomical, physiological, and immunological similarities to humans [2, 3]. Enhancing the efficiency of genome editing in porcine embryos remains a key objective for advancing xenotransplantation and disease-modeling applications.

Various chemical and physical methods have been developed to improve the intracellular delivery of CRISPR components. Cationic lipids, such as lipofectamine, form lipoplexes with nucleic acids, neutralizing their negative charge and increasing lipophilicity, thereby promoting cellular uptake via endocytosis or membrane fusion [4–6]. This process, known as lipofection, enables efficient delivery of DNA or RNA into cells by reducing electrostatic repulsion between the cargo and the plasma membrane.

In contrast, electroporation (EP) relies on transient electrical pulses to create temporary pores in the cell membrane, enabling passive diffusion or electrically driven entry of exogenous macromolecules, including CRISPR/Cas9 ribonucleoprotein (RNP) complexes [7]. While both methods have been individually optimized, their combined use may exploit complementary mechanisms: lipid-mediated membrane affinity and EP-induced membrane permeability.

We hypothesized that EP destabilizes the membranes of porcine zygotes, thereby enhancing the interaction of cationic lipid-coated RNPs with the lipid bilayer. This cooperative effect could facilitate more efficient uptake and intracellular release of RNP complexes, thereby improving gene-editing outcomes.

From a biomedical perspective, disrupting the *B4GALNT2* gene, which encodes β-1,4-N-acetyl-galactosaminyltransferase 2, responsible for xenoantigen synthesis, is critical for reducing immune rejection in xenotransplantation [8]. Similarly, targeted inactivation of the growth hormone receptor (*GHR*) gene, which is mutated in humans and causes Laron syndrome, can yield pigs with proportionally reduced organ size, aligning porcine organ dimensions with human physiology for potential transplantation applications [9].

Despite the remarkable success of CRISPR/Cas9 in generating genetically modified livestock, its efficiency in porcine embryos remains suboptimal due to limitations in the intracellular delivery of Cas9-gRNA RNP complexes. Conventional microinjection offers precise delivery but is technically demanding, low-throughput, and can compromise embryo viability. EP has emerged as a simpler and scalable alternative for porcine zygotes; however, its editing efficiency varies widely and is often constrained by inconsistent membrane permeability and incomplete cytoplasmic diffusion of RNPs.

Chemical transfection methods, such as lipofection, have been shown to facilitate the uptake of macromolecules by encapsulating them within cationic lipid vesicles. Although lipofection enables efficient gene delivery in somatic cells and embryos of smaller animals, its application in porcine zygotes is limited by the need to remove the zona pellucida (ZP) and by concerns about lipid-associated cytotoxicity. Few studies have attempted to combine chemical and physical approaches to leverage their complementary mechanisms, lipid-mediated fusion, and EP-induced membrane permeabilization, for enhanced RNP transfer. To date, there is a lack of systematic evaluation of whether packaging CRISPR/Cas9 RNPs in cationic lipids can synergistically improve EP-mediated gene editing in porcine embryos while maintaining normal embryonic development.

This study aimed to develop and evaluate a combined lipofection-EP strategy for efficient CRISPR/Cas9-mediated genome editing in porcine zygotes. Specifically, we investigated whether packaging RNPs in lipofectamine could enhance their delivery and mutagenesis efficiency when introduced by EP. Using two representative target genes, *B4GALNT2*, a key xenoantigenic gene relevant to xenotransplantation, and *GHR*, a gene associated with growth regulation, we compared the effects of different delivery sequences, including EP alone, EP with lipofectamine-packaged RNPs (EPL), and sequential lipofection before or after EP. The outcomes were assessed based on embryo development, mutation rate, and editing efficiency.

Through this approach, we sought to (1) determine the feasibility and safety of using cationic lipid-packaged RNPs in porcine zygotes, (2) evaluate whether sequential dual-delivery can enhance genome-editing efficiency, and (3) establish a reproducible, low-toxicity workflow suitable for generating gene-edited pigs for biomedical and xenotransplantation research.

## MATERIALS AND METHODS

### Ethical approval

This study did not involve the use of live animals for experimental purposes. Porcine ovaries were obtained as by-products from a local slaughterhouse, where animals were slaughtered for commercial meat production and not specifically for this research. Therefore, no ethical approval for animal experimentation was required.

All experimental procedures involving the collection and handling of biological materials were conducted in strict accordance with the ethical standards and guidelines approved by the Institutional Animal Care and Use Committee of Tokushima University, Japan. The study also adhered to the principles outlined in the Animal Research: Reporting of *In Vivo* Experiments (ARRIVE) 2.0 guidelines and the Directive 2010/63/EU for the protection of animals used for scientific purposes.

### Study period and location

All experiments were performed between April and June 2024 at the Bio-Innovation Research Center, Tokushima University, Japan.

### General procedures for the *in vitro* production of porcine embryos

#### Oocyte collection and in vitro maturation (IVM)

The procedures for oocyte collection, IVM, *in vitro* fertilization (IVF), and embryo culture were performed as described by Lin *et al*. [10]. Briefly, ovaries from prepubertal crossbred gilts (Landrace × Large White × Duroc) were collected from a local slaughterhouse and transported to the laboratory in physiological saline maintained at 30°C.

Using a surgical blade, cumulus–oocyte complexes (COCs) that exhibited uniformly dark-pigmented ooplasm and intact cumulus cell masses were collected from follicles. Approximately 50 COCs were cultured in 500 µL of maturation medium consisting of tissue culture medium 199 with Earle’s salts (TCM 199; Thermo Fisher Scientific, Waltham, MA, USA; 11150059) supplemented with 10% (v/v) porcine follicular fluid, 0.6 mM cysteine (Sigma-Aldrich, St. Louis, MO, USA; C7352), 50 µg/mL gentamicin (Sigma-Aldrich; G3632), 50 µM sodium pyruvate (Sigma-Aldrich; P5280), 50 µM β-mercaptoethanol (Sigma-Aldrich; M3148), 2 mg/mL D-sorbitol (Sigma-Aldrich; S7547), 10 IU/mL equine chorionic gonadotropin (Kyoritsu Seiyaku, Tokyo, Japan), and 10 IU/mL human chorionic gonadotropin (Kyoritsu Seiyaku).

The maturation medium was sterilized by filtration using a 0.22 µm filter. Initially, COCs were cultured in hormone-supplemented IVM medium for 22 h in 4-well dishes (Nunc A/S, Roskilde, Denmark; 176740), followed by an additional 22 h in hormone-free IVM medium at 39°C in a humidified atmosphere of 5% CO_2_ in air (MCO-5AC-PE; PHC Corp., Tokyo, Japan). Humidity was maintained by a water pan inside the incubator, and gas composition was automatically regulated and continuously monitored by the internal control system to ensure stable culture conditions.

#### IVF and embryo culture

During IVF, frozen-thawed spermatozoa were placed in 5 mL of porcine fertilization medium (PFM; IFP1020P; Research Institute for the Functional Peptides Co., Yamagata, Japan) and centrifuged at 500 × *g* for 5 min to wash. The sperm pellets were resuspended in PFM and adjusted to a final concentration of 1 × 106 sperm/mL. Approximately 50 mature oocytes were transferred to 500 µL of PFM containing sperm and co-incubated for 5 h.

After coincubation, inseminated oocytes were denuded and cultured in porcine zygote medium (PZM-5; IFP0410P; Research Institute for the Functional Peptides Co.) covered with mineral oil (M8410; Sigma-Aldrich) until gene editing was performed. The zygotes were cultured in PZM-5 for 3 days following treatment. Subsequently, all cleaved embryos were transferred to porcine blastocyst medium (PBM; IFP1030P; Research Institute for the Functional Peptides Co.) and cultured for an additional 4 days to evaluate their development to the blastocyst stage and determine genotypes. All embryo manipulations were conducted under sterile conditions within a clean bench to prevent contamination.

### Experimental interventions

#### ZP removal

Since ZP removal is required to transfect RNPs using the lipofection method [11], the ZP of zygotes was removed before both transfection and EP. Zygotes were treated with 0.5% (w/v) actinase-E (Kaken Pharmaceutical Co., Tokyo, Japan; 650133) in Dulbecco’s phosphate-buffered saline (Thermo Fisher Scientific; 14190144) for 20–30 s. They were then transferred to PZM-5 without actinase-E and completely freed from their ZP by gentle pipetting. Following removal, ZP-free zygotes were cultured individually in PZM-5 and PBM in 25-well dishes (ART Culture Dish; Nipro Co., Osaka, Japan; 87435), as described by Lin *et al*. [12].

#### EP using RNPs (EP)

EP was performed according to Tanihara *et al*. [13] with slight modifications. An electrode (LF501PT1-20; BEX, Tokyo, Japan) was positioned beneath a stereomicroscope and connected to a CUY21EDIT II electroporator (BEX). Approximately 50 ZP-free zygotes were rinsed with Opti-MEM I solution (Invitrogen Co., Carlsbad, CA, USA; 31985062) and aligned between electrodes on a chamber slide containing 10 µL of Nuclease-Free Duplex Buffer (IDT; Integrated DNA Technologies, IA, USA; 11010301).

This buffer included 100 ng/µL Cas9 protein (Guide-it Recombinant Cas9, Takara Bio, Shiga, Japan; 632641) and 100 ng/µL gRNAs (Alt-R CRISPR crRNAs and tracrRNA; IDT) targeting B4GALNT2 (5′-ATGTGACGCCTTCGGG CATC-3′; 109047933) or GHR (5′-CTGTTGACCTTGGCAGTGGC-3′; 109047932). Zygotes were electroporated using five 1-ms pulses at 25 V. Following EP, ZP-free zygotes were cultured individually in PZM-5 and PBM as described above.

#### Transfection through lipofectamine (TL)

Lipofectamine 2000 (Thermo Fisher Scientific; 11668019) was used for transfection as described by Lin *et al*. [10]. The transfection mixture was prepared by combining 2 µL of lipofectamine 2000 with 8 µL of Nuclease-Free Duplex Buffer containing pre-formed RNPs. The RNP complexes were generated by mixing 300 ng/µL Cas9 protein with 100 ng/µL gRNAs targeting either *B4GALNT2* or *GHR*, yielding a total volume of 20 µL.

After 15 min of incubation, the mixture was added to 180 µL of PZM-5 containing 50 ZP-free zygotes for transfection. Following 5 h of incubation, ZP-free zygotes were washed and cultured individually in PZM-5 and PBM, as described above.

#### EP with lipofectamine-packaged RNPs (EPL)

EPL refers to EP performed using RNPs packaged with lipofectamine 2000. The EP mixture was prepared by combining 1 µL of lipofectamine 2000 with 4 µL of RNP-containing Nuclease-Free Duplex Buffer. These RNPs were composed of 100 ng/µL Cas9 protein and 100 ng/µL gRNAs targeting either *B4GALNT2* or *GHR*, resulting in a total volume of 10 µL.

The prepared solution was used for EP as described above. After EP, the ZP-free zygotes were cultured individually in PZM-5 and PBM under identical conditions.

#### Combination treatments (TL + EPL and EPL + TL)

Two combination protocols were evaluated:

1. TL + EPL: ZP-free zygotes were first transfected with lipofectamine-RNP complexes for 5 h (5 h post-IVF), followed by EPL.
2. EPL + TL: Zygotes were first electroporated with lipofectamine-packaged RNPs 10 h after IVF, followed by lipofection for 5 h with RNP-containing lipofectamine 2000.

### Experimental design and treatment groups

**Figure 1:**
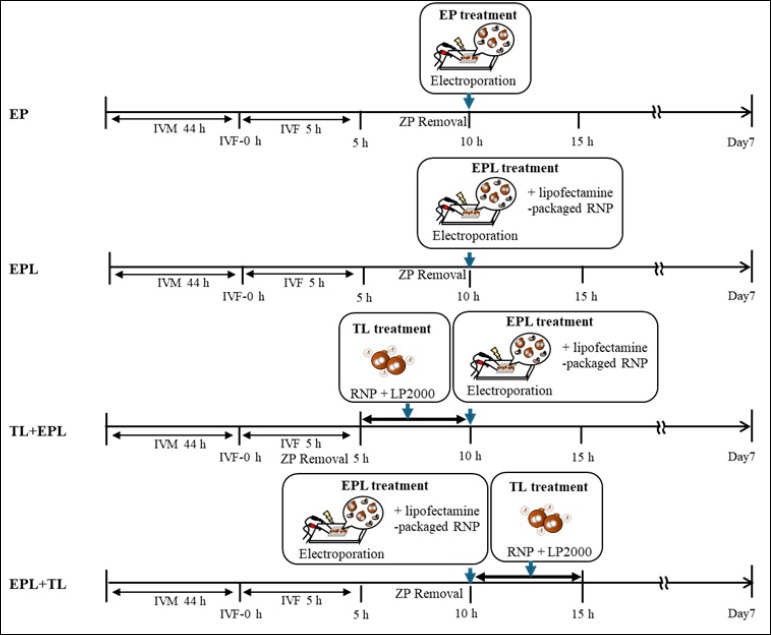
Schematic of the experimental design. Zona pellucida-free zygotes were edited with gRNA targeting either beta-1,4-N-acetyl-galactosaminyl transferase 2 (*B4GALNT2*) or growth hormone receptor (*GHR*) genes by combining EP or transfection. IVM = In vitro maturation, IVF = In vitro fertilization, LP2000 = Lipofectamine 2000, RNP = Cas9-gRNA ribonucleoprotein complex, EP = Electroporation using RNPs, TL = Transfection through lipofectamine, EPL = Electroporation with lipofectamine-packaged RNPs.

The experimental design is schematically illustrated in Figure 1. ZP-free zygotes were edited using gRNAs targeting either the *B4GALNT2* or *GHR* genes through different delivery strategies. In a previous study by Le *et al*. [14], zygotes lacking RNPs and sham-electroporated controls showed cleavage and blastocyst development comparable to those of untreated embryos. Therefore, the EP group (RNPs without lipofectamine) was used as an internal reference for baseline editing and developmental performance.

The treatment groups were as follows:

1. EP group: ZP-free zygotes electroporated with RNPs without lipofectamine 10 h post-IVF (control).
2. EPL group: ZP-free zygotes electroporated with RNPs packaged in lipofectamine 10 h post-IVF.
3. TL + EPL group: ZP-free zygotes transfected with lipofectamine-RNPs 5 h post-IVF for 5 h, then electroporated with lipofectamine-packaged RNPs.
4. EPL + TL group: ZP-free zygotes electroporated with lipofectamine-packaged RNPs 10 h post-IVF, then transfected for 5 h with lipofectamine-RNPs.

### Assessment of embryonic development

After EP and transfection, zygotes were cultured individually for 7 days to evaluate blastocyst formation, mutation rate, and mutation efficiency. Blastocyst formation was defined morphologically as embryos reaching the blastocyst stage by day 7, characterized by a fluid-filled cavity and a visible inner cell mass.

For each treatment, 40–50 zygotes were analyzed per replicate, and five independent biological replicates were performed, yielding 200–250 embryos per group.

### Genotyping and mutational analysis

Genomic DNA from individual blastocysts was extracted by boiling in 50 mM NaOH (Wako Pure Chemical Industries Ltd.; 19418865), followed by neutralization. Polymerase chain reaction (PCR) amplification was conducted using KOD One PCR Master Mix (Toyobo, Osaka, Japan; KMM201) with specific primers:

1. B4GALNT2: Forward 5′-TAGGGGGAAAAACACACTGG-3′ and Reverse 5′-CACCCTCGGGAATGAGTAGA-3′.
2. GHR: Forward 5′-CCCACCGGAAGTAGCATTTA-3′ and Reverse 5′-ACAACACTCCCGGAAACATC-3′.

PCR products were separated through agarose gel electrophoresis and purified using the FastGene Gel/PCR Extraction Kit (Nippon Genetics, Tokyo, Japan; FG91302). Sequencing was performed with the BigDye Terminator Cycle Sequencing Kit v3.1 (Thermo Fisher Scientific) and an ABI 3500 Genetic Analyzer (Applied Biosystems, Foster City, CA, USA).

The tracking of indels by decomposition (TIDE) bioinformatics tool [15] was employed to assess indel composition. Blastocysts were classified as biallelic mutants (wild-type ≤5%), mosaics (mixed indel and wild-type >5%), or wild-type (<5% indels). The mutation rate was defined as the ratio of gene-edited blastocysts to the total number of sequenced blastocysts, and mutation efficiency was defined as the proportion of indel events in mutant blastocysts.

### Statistical analysis

Data for embryo development and mutation efficiency were analyzed using analysis of variance, followed by Fisher’s protected least significant difference test (STATVIEW, version 5.0; Abacus Concepts, Inc., Berkeley, CA, USA). Proportion data were arcsine-transformed to stabilize variance and approximate normality.

Normality of distribution was assessed using the Jarque–Bera test. The proportions of mutated blastocysts were compared using the Chi-square test with Yates’ correction. Statistical significance was accepted at p < 0.05.

## RESULTS

### Embryonic development after gene editing

The blastocyst formation rates did not significantly differ among the treatment groups for zygotes edited with gRNA targeting *B4GALNT2* (Table 1). All groups, including EP alone, EPL, and combined treatments, showed comparable cleavage and blastocyst development, indicating that lipofectamine packaging and EP did not negatively affect embryo viability.

**Table 1:** Embryo development and mutation efficiency of zona pellucida-free zygotes edited by Cas9-gRNA RNPs targeting *the B4GALNT2* gene by electroporation or transfection.

Group*	A	B	C	D	E	F	G
EP	225	30 (13.0 ± 3.3)	30	0 (0)ᵃ	0 (0)ᵃ	0 (0)	–
EPL	231	66 (28.4 ± 6.4)	30	0 (0)ᵃ	0 (0)ᵃ	0 (0)	–
TL + EPL	203	29 (13.8 ± 5.2)	27	5 (18.5)ᵇ	5 (18.5)ᵇ	0 (0)	20.1 ± 8.0
EPL + TL	215	48 (21.7 ± 5.5)	29	2 (6.9)ᵃᵇ	2 (6.9)ᵃᵇ	0 (0)	7.9 ± 1.3

A: Number of zygotes examined, B: Number (%) of embryos developed to blastocysts, C: Number of blastocysts examined, D: Number (%) of gene-edited embryos – *Total*, E: Number (%) of gene-edited embryos – *Mosaic*, F: Number (%) of gene-edited embryos – *Biallelic*, G: Mutation efficiency, * Group descriptions, ** Blastocyst development rate, *** Gene-edited embryos classification, **** Mutation efficiency: mean ± SD

*EP = ZP-free zygotes were electroporated with Cas9-gRNA RNPs without lipofectamine, EPL = ZP-free zygotes were electroporated with Cas9-gRNA RNPs packaged in lipofectamine, TL + EPL = ZP-free zygotes were transfected with lipofectamine containing Cas9-gRNA RNPs and subsequently electroporated with Cas9-gRNA RNPs packaged in lipofectamine, EPL + TL = ZP-free zygotes were electroporated with Cas9-gRNA RNPs packaged in lipofectamine and subsequently transfected with lipofectamine containing Cas9-gRNA RNPs. **Percentages are expressed as the mean ± SEM, which indicates variability among five independent replicates. ***Percentages of mutation were calculated by dividing the number of gene-edited blastocysts by the number of examined blastocysts. Total, all mutants; mosaic, mosaic mutants; biallelic, biallelic mutants. ****Mutation efficiency represents the proportion (mean ± SEM) of indel mutation events in mutant blastocysts as determined by TIDE analysis. Different superscript letters in the same column indicate groups with significant differences (p < 0.05).

Similarly, for gRNA targeting *GHR*, blastocyst formation rates remained consistent across all experimental groups (Table 2). These findings demonstrate that the use of lipofectamine, either alone or in combination with EP, was not cytotoxic to porcine embryos and did not compromise their developmental competence to the blastocyst stage.

**Table 2:** Embryo development and mutation efficiency of zona pellucida (ZP)-free zygotes edited by Cas9-gRNA RNPs targeting *the GHR* gene by electroporation or transfection.

Group*	No. of zygotes examined	No. (%) embryos developed to blastocysts**	No. of blastocysts examined	Number (%) of gene-edited embryos***			Mutation efficiency****

				Total	Mosaic	Biallelic	
EP	214	25 (11.7 ± 3.7)	25	13 (52.0)^a^	8 (32.0)^a^	5 (20.0)	62.4 ± 9.8
EPL	237	51 (21.3 ± 6.0)	29	22 (75.9)^ab^	20 (69.0)^b^	2 (6.9)	54.8 ± 6.8
TL + EPL	212	52 (23.8 ± 5.2)	28	26 (92.9)^b^	18 (64.3)^b^	8 (28.6)	65.4 ± 6.1
EPL + TL	224	51 (23.0 ± 5.2)	30	17 (56.7)^a^	11 (36.7)^a^	6 (20.0)	63.9 ± 8.0

* EP = ZP-free zygotes were electroporated with Cas9-gRNA RNPs without lipofectamine. EPL = ZP-free zygotes were electroporated with Cas9-gRNA RNPs packaged in lipofectamine. TL + EPL = ZP-free zygotes were transfected with lipofectamine containing Cas9-gRNA RNPs and subsequently electroporated with Cas9-gRNA RNPs packaged in lipofectamine. EPL + TL = ZP-free zygotes were electroporated with Cas9-gRNA RNPs packaged in lipofectamine and subsequently transfected with lipofectamine containing Cas9-gRNA RNPs.

** Percentages are expressed as the mean ± SEM, which indicates variability among five independent replicates.

*** Percentages of mutation were calculated by dividing the number of gene-edited blastocysts by the number of examined blastocysts. Total, all mutants; mosaic, mosaic mutants; biallelic, biallelic mutants.

**** Mutation efficiency represents the proportion (mean ± SEM) of indel mutation events in mutant blastocysts as determined by TIDE analysis. Different superscript letters in the same column indicate groups with significant differences (p < 0.05). GHR = Growth hormone receptor

### Mutation rate and efficiency in *B4GALNT2*-targeted embryos

No mutant blastocysts were obtained from embryos electroporated with RNPs alone (EP) or from those electroporated with lipofectamine-packaged RNPs (EPL). In contrast, the TL + EPL group (transfection followed by EP) produced a significantly higher total and mosaic mutation rates compared with both the EP and EPL groups (p < 0.05). However, mutation efficiency, defined as the proportion of indel events within mutant embryos, did not differ significantly among the groups.

These results suggest that the sequential combination of lipofection and EP enhances the likelihood of introducing CRISPR-mediated mutations in porcine zygotes, even though the overall indel frequency within edited embryos remains unchanged.

### Mutation rate and efficiency in *GHR*-targeted embryos

For embryos edited with gRNA targeting *GHR*, the mosaic mutation rate was significantly higher in the EPL group than in the EP group (p < 0.05). The TL + EPL treatment further increased both the total and mosaic mutation rates relative to the EP group (p < 0.05), while mutation efficiency remained statistically similar among all groups.

These findings indicate that pre-EP transfection with lipofectamine-packaged RNPs (TL) effectively enhances genome-editing frequency without impairing embryo development. The improved outcomes in the TL + EPL group highlight the synergistic potential of combining chemical and physical delivery methods to improve CRISPR/Cas9-mediated mutagenesis in porcine zygotes.

## DISCUSSION

### Enhancing CRISPR/Cas9 delivery efficiency in porcine zygotes

Improving the efficiency of gene delivery into porcine zygotes and embryos has become a major focus in genome-editing research. In this study, we investigated whether packaging RNPs in cationic lipids (lipofectamine) could synergistically enhance EP-mediated gene editing while maintaining embryo viability. Such improvements are especially relevant for generating genetically modified pigs used in xenotransplantation and as biomedical models for human diseases.

Our findings demonstrated that the inclusion of lipofectamine during EP did not adversely affect the developmental competence of zygotes, either when applied alone or in combination with transfection. This suggests that lipofectamine has minimal cytotoxicity under the experimental conditions used. The combined EP transfection approach also maintained normal embryonic development, consistent with previous observations by Takebayashi *et al*. [11] and Lin *et al*. [16].

### Embryo viability and effects of ZP removal

Although lipofection-based delivery systems can sometimes induce cytotoxicity in oocytes [17], no adverse effects were observed in this study. The ZP must be removed to facilitate lipofection of RNPs; however, ZP removal can negatively influence developmental potential [11]. To minimize this effect, we cultured ZP-free embryos individually in 25-well dishes as described by Lin *et al*. [12]. This culture approach likely reduced potential stress associated with ZP removal, thereby supporting normal embryonic development. Overall, lipofectamine packaging did not impair blastocyst formation, indicating that the treatment was well tolerated by porcine zygotes.

### Synergistic interaction between lipofection and EP

While lipofectamine-packaged RNPs alone did not significantly increase mutation rates during EP, the addition of a pre-EP transfection step with RNPs markedly improved total and mosaic mutation rates. This represents a significant improvement over EP alone, which typically yields modest mutation rates in porcine embryos [14].

For *B4GALNT2*-targeted embryos, EP alone failed to induce detectable mutations, whereas introducing lipofectamine-mediated transfection before or after EP successfully produced mutants. For *GHR*-targeted embryos, the TL + EPL treatment, lipofection followed by EP, resulted in the highest total and mosaic mutation rates. This improvement may be explained by the initial adsorption of lipid-RNP complexes to the zygote membrane during transfection. Subsequent EP likely destabilized the membrane, promoting the fusion of lipid complexes and enhancing intracellular RNP uptake. Such a synergistic mechanism warrants further mechanistic validation in future CRISPR delivery studies.

### Ineffectiveness of EP with lipofectamine-packaged RNPs

In contrast, EP using pre-formed lipofectamine-RNP complexes (EPL group) did not yield significant improvements in mutation rate or efficiency. This reduced effect may be due to the formation of large nanocomplexes, which can limit transport through EP-induced membrane pores. Optimizing the size and surface charge of these complexes may be crucial for improving delivery efficiency in future EP-based systems.

Similarly, the post-EP transfection (EPL + TL) approach was ineffective, likely because membrane pores generated by EP resealed rapidly. As pore resealing is essential for maintaining cell viability [18, 19], this phenomenon may have reduced subsequent RNP uptake. These observations suggest that the sequence and timing of dual delivery are critical determinants of genome-editing success.

## CONCLUSION

This study demonstrated that combining lipofection and EP provides a synergistic, non-toxic strategy to improve CRISPR/Cas9-mediated genome editing in porcine zygotes. The packaging of Cas9-gRNA RNP complexes in lipofectamine did not adversely affect embryonic development, as the cleavage and blastocyst formation rates were comparable among all treatment groups. However, sequential transfection of lipofectamine-packaged RNPs before EP (TL + EPL) significantly increased both the total and mosaic mutation rates compared with EP alone, indicating enhanced intracellular delivery and editing efficiency. In contrast, EP with pre-formed lipofectamine-RNP complexes or transfection after EP did not yield comparable improvements, likely due to limited membrane permeability or rapid pore resealing.

The findings provide a practical and reproducible workflow for introducing CRISPR/Cas9 RNPs into porcine zygotes, offering a simpler alternative to microinjection-based methods. The combined lipofection-EP approach enhances genome-editing efficiency while maintaining high embryo viability, making it particularly suitable for producing genetically modified pigs intended for xenotransplantation, human disease modeling, and agricultural biotechnology.

A major strength of this work is the systematic evaluation of both chemical (lipofection) and physical (EP) gene-delivery methods within a single experimental framework. The design allowed for a direct comparison of sequence-dependent effects (before vs. after EP) and revealed the mechanistic importance of delivery order in achieving higher mutation rates. Furthermore, the study confirmed the safety of lipofectamine use in ZP-free embryos under optimized culture conditions.

The study was conducted under *in vitro* conditions only, and post-implantation or live-birth outcomes were not evaluated. Additionally, the investigation focused on mutation frequency and efficiency without assessing off-target effects or long-term developmental performance. The nanoparticle size and biophysical properties of lipofectamine-RNP complexes were not characterized, which could further clarify delivery efficiency and cellular uptake dynamics.

Future research should include embryo transfer experiments to evaluate developmental competence and germline transmission of mutations. Characterization of RNP-lipid nanostructures, optimization of pulse parameters, and incorporation of high-fidelity Cas9 variants or microfluidic-assisted delivery could further refine this approach. Expanding the methodology to other large-animal species would enhance its translational potential for biomedical engineering and precision livestock breeding.

In conclusion, the sequential use of lipofection followed by EP represents an efficient, low-toxicity, and scalable platform for genome-editing in porcine embryos. This dual-delivery system not only advances the precision of gene editing workflows in livestock research but also supports the broader development of next-generation animal models for regenerative medicine and organ transplantation.

## DATA AVAILABILITY

All data generated are included in the manuscript.

## AUTHORS’ CONTRIBUTIONS

QL: Performed the experiments and drafted the manuscript. TO: Designed the study and revised the manuscript. OSW, TT, and KC: Supervised and reviewed the manuscript. ZN, MH, AN, YN, and MN: Performed experimental assistance and statistical analyses. FT: Performed all experiments and reviewed the manuscript. All authors have read and approved the final version of the manuscript.
